# 1-Hour Post-OGTT Glucose Improves the Early Prediction of Type 2 Diabetes by Clinical and Metabolic Markers

**DOI:** 10.1210/jc.2018-01828

**Published:** 2018-11-15

**Authors:** Gopal Peddinti, Michael Bergman, Tiinamaija Tuomi, Leif Groop

**Affiliations:** 1 VTT Technical Research Center of Finland Ltd, Espoo, Finland; 2 NYU School of Medicine, Department of Medicine, Division of Diabetes, Endocrinology and Metabolism, NYU Langone Diabetes Prevention Program, New York, New York; 3 Folkhälsan Research Center, Helsinki, Finland; 4 Abdominal Center, Endocrinology, Helsinki University Central Hospital; Research Program for Diabetes and Obesity, University of Helsinki, Helsinki, Finland; 5 Institute for Molecular Medicine Finland (FIMM), University of Helsinki, Helsinki, Finland; 6 Lund University Diabetes Centre, Department of Clinical Sciences, Lund University, Skåne University Hospital, Malmö, Sweden

## Abstract

**Context:**

Early prediction of dysglycemia is crucial to prevent progression to type 2 diabetes. The 1-hour postload plasma glucose (PG) is reported to be a better predictor of dysglycemia than fasting plasma glucose (FPG), 2-hour PG, or glycated hemoglobin (HbA1c).

**Objective:**

To evaluate the predictive performance of clinical markers, metabolites, HbA1c, and PG and serum insulin (INS) levels during a 75-g oral glucose tolerance test (OGTT).

**Design and Setting:**

We measured PG and INS levels at 0, 30, 60, and 120 minutes during an OGTT in 543 participants in the Botnia Prospective Study, 146 of whom progressed to type 2 diabetes within a 10-year follow-up period. Using combinations of variables, we evaluated 1527 predictive models for progression to type 2 diabetes.

**Results:**

The 1-hour PG outperformed every individual marker except 30-minute PG or mannose, whose predictive performances were lower but not significantly worse. HbA1c was inferior to 1-hour PG according to DeLong test *P* value but not false discovery rate. Combining the metabolic markers with PG measurements and HbA1c significantly improved the predictive models, and mannose was found to be a robust metabolic marker.

**Conclusions:**

The 1-hour PG, alone or in combination with metabolic markers, is a robust predictor for determining the future risk of type 2 diabetes, outperforms the 2-hour PG, and is cheaper to measure than metabolites. Metabolites add to the predictive value of PG and HbA1c measurements. Shortening the standard 75-g OGTT to 1 hour improves its predictive value and clinical usability.

## Teaser

We evaluated clinical risk factors, metabolites, HbA1c, and plasma glucose levels during an OGTT. The 1-hour glucose level either alone or together with metabolites robustly predicted type 2 diabetes.

Because type 2 diabetes may be a preventable disease (1), early prediction is crucial. Fasting plasma glucose (FPG) and 2-hour plasma glucose (PG) after the 75-g oral glucose tolerance test (OGTT) are used to assess the risk of developing type 2 diabetes. Several recent studies have demonstrated that 1-hour postload PG is a better predictor of dysglycemia than FPG, 2-hour PG, or glycated hemoglobin (HbA1c) (2) and that 1-hour PG is predictive of not only type 2 diabetes but also cardiovascular disease and mortality (3–7). Furthermore, the CATAMERI study found that people with normal glucose tolerance having an elevated 1-hour PG level ≥8.6 mmol/L were at higher risk for developing chronic kidney disease (8), nonalcoholic fatty liver disease (9), increased vascular stiffness (10), and early carotid atherosclerosis (11). An elevated 1-hour PG level was also associated with decreased insulin (INS) clearance (12), INS sensitivity, and reduced *β*-cell function (13), an unfavorable inflammatory profile (14), and whole blood viscosity (15).

Meanwhile, metabolomics has also provided a rich source of predictive markers for future progression to type 2 diabetes (16, 17). A number of prospective studies identified metabolic biomarkers including branched chain and aromatic amino acids, sugars and carbohydrates, and lipids (phospholipids, triglycerides) to be associated with the incidence of prediabetes (*ie,* impaired glucose tolerance, impaired fasting glucose, INS resistance, or impaired INS sensitivity) and type 2 diabetes (17). Using mass spectrometry-based metabolomics in a Botnia Prospective Study (BPS) cohort, we previously performed a systematic assessment of metabolites in predicting future progression to type 2 diabetes and identified seven metabolic markers that provided the best predictive model in combination with clinical risk factors (18). These metabolic markers included glucose, mannose, *α*-hydroxybutyrate (AHB), *α*-tocopherol, bradykinin-hydroxyproline ([Hyp3]-BK), and the unknown metabolites X-12063 and X-13435, the latter of which was identified as 10:1 carnitine during this study.

Given the predictive performance of OGTT-derived PG measurements and metabolites, we hypothesized that combining PG measurements with metabolites would further improve prediction of future risk for type 2 diabetes. Therefore, we performed a systematic assessment of the predictive performance of PG levels measured at different time points during the OGTT, HbA1c, metabolic markers, and clinical risk factors such as age, sex, body mass index (BMI), and familial history (FH) of type 2 diabetes, and we considered the clinical translatability of our findings. Although the predictive performance of PG values and metabolites has been studied previously, the novelty of our contribution is in providing a systematic assessment of their joint predictive performance for future progression to type 2 diabetes.

## Materials and Methods

### Study population

The BPS, initiated in 1990 on the west coast of Finland, includes a cohort of 2770 nondiabetic people followed for 10 years (median 7.7 years), 150 of whom developed type 2 diabetes (19). In the current study, we included a BPS subpopulation of 543 participants, including 146 who progressed to type 2 diabetes (called progressors) by the end of the follow-up period [Table D1 in (20)], in whom serum metabolomics data were available (18). The control sample of 397 participants in the current study was randomly chosen from among the 2620 nonprogressors.

Helsinki University Central Hospital approved the study protocols. All participants gave informed consent to their participation in the study.

### OGTT

All participants underwent a 75-g OGTT after a 12-hour overnight fast. Participants were instructed to eat and exercise normally for 3 days before the OGTT. PG levels were measured at 0, 30, 60, and 120 minutes during the OGTT via the glucose oxidase method (Beckman Glucose Analyzer, Beckman Instruments, Fullerton, CA). Serum INS levels during the OGTT were measured by RIA (Linco; Pharmacia, Uppsala, Sweden) or an enzyme immunoassay (DAKO, Cambridgeshire, UK); the correlation between the methods after conversion was 0.98 (*P* < 0.0001).

### Clinical risk factors and diagnostic criteria

Sex, age, BMI, and FH were recorded at baseline. HbA1c was measured via high-performance liquid chromatography. The World Health Organization criteria (21) were used to define impaired fasting glucose (IFG; 6.1 mmol/L ≤ FPG < 7.0 mmol/L and 2-hour PG < 7.8 mmol/L) and impaired glucose tolerance (IGT; FPG < 7.0 mmol/L and 7.8 mmol/L ≤ 2-hour PG < 11.1 mmol/L) [Table D1 in (20)]. With this, we determined that 86 participants had IFG, 112 had IGT, and 324 had neither at the baseline.

The World Health Organization diagnostic criteria (FPG ≥7.0 mmol/L or 2-hour PG ≥11.1 mmol/L) were used to define whether a participant had progressed to type 2 diabetes based on a 75-g OGTT during the final follow-up visit for the participant.

### Statistical analysis

In this study, participants with missing measurements for any variable were excluded from all analyses involving that particular variable. To test the association of a variable with progression to type 2 diabetes, the Fisher exact test was used for categorical variables, and the Welch *t* test was used for continuous variables [Table D1 in (20)]. Correlation between continuous variables was tested with the Pearson correlation test [Tables D2 and D3 in (20)].

### Metabolomics and identification of metabolic markers

Ferrannini *et al.* (22) applied a clinically validated targeted metabolomics assay to measure two metabolites—AHB and 1-linoleoyl glycerophosphocholine—in a larger BPS subpopulation (n = 2580) than the current study (n = 543). In contrast, the current study used a combination of global untargeted and targeted metabolomics approaches and measured 568 metabolites. Fasting serum samples collected at baseline were used for metabolomic profiling performed at Metabolon Inc. (Durham, NC). Samples were prepared with the single extraction method and were subjected to untargeted and targeted metabolomics, via ultra-high-performance liquid chromatography or gas chromatography coupled with mass spectrometry and isotope-dilution ultra-high-performance liquid chromatography coupled with tandem mass spectrometry, respectively (18). Metabolites were identified by automated spectral comparison with a standard library, and missing values were imputed with minimum nonmissing measurement. The targeted and untargeted metabolomics data were standardized to zero mean and unit variance per metabolite and combined into a single data matrix containing 568 metabolite measurements from 543 samples.

A metabolic marker panel consisting of glucose, mannose, AHB, [Hyp3]-BK, *α*-tocopherol, and the unknown metabolites X-13435 and X-12063 was derived with a machine learning–based feature selection approach, as described previously (18). Using updated mass spectral identification libraries, Metabolon Inc. identified X-13435 as 10:1 carnitine during this study.

### Predictive models

We evaluated predictive performances of various combinations (n = 1527) of clinical risk factors, PG, and INS levels during OGTT, HbA1c, and six metabolic markers (mannose, AHB, [Hyp3]-BK, *α*-tocopherol, 10:1 carnitine, and X-12063) via machine learning–based predictive models for future progression to type 2 diabetes. Briefly, a regularized least squares regression approach (23) was used to build binary classifiers via the repeated nested cross-validation described in (18) with 100 repetitions. Average scores from the 100 predictions were calculated to construct the receiver operating characteristic (ROC) curve. The optimal classification threshold for the prediction score was determined from the ROC curve based on the F-index of sensitivity and specificity values. That is, the harmonic mean of sensitivity and specificity was calculated for each threshold value on the ROC curve, and the value corresponding to the maximum harmonic mean was selected as the optimal cutoff. The binary classification accuracy, sensitivity, specificity, negative predictive value (NPV), and positive predictive value (PPV) were calculated at the optimal cutoff. The 95% CI for the area under the receiver operating characteristic curve (AUC) was computed via DeLong’s method with 2000 stratified bootstrap replicates (24, 25). Predictive performance of each model was compared with that of 1-hour PG via DeLong’s test for the comparison of correlated ROC curves (24). To address the multiple testing problem arising from the large number (n = 1526) of comparisons, *q*-values were calculated with the positive false discovery rate approach (26). The *q*-values were rounded to two decimal digits, and the results with *q* < 0.05 after the rounding were considered statistically significant.

## Results

### Identification of an unknown metabolite

We previously identified a panel of seven metabolic markers—glucose, mannose, AHB, [Hyp3]-BK, *α*-tocopherol, and the unidentified metabolites X-13435 and X-12063—by using predictive modeling of serum metabolomics data (18). Reidentification during the current study with an updated Metabolon mass spectral identification library identified X-13435 as 10:1 carnitine. Therefore, in this study we refer to X-13435 as 10:1 carnitine.

### Correlations between PG measurements

The PG levels significantly correlated with each other. The consecutive time points showed higher correlations with each other than with time points further apart. The FPG showed higher correlation with 30-minute PG, the 30-minute PG with 1-hour PG, and the 2-hour PG with 1-hour PG than with other time points [Table D2 in (20)]. The 30-minute PG and 1-hour PG pair showed the highest correlation of all PG pairs. HbA1c showed higher correlation with post-OGTT PG levels than with FPG.

### Correlation between glucose measurements and metabolic markers

**Figure 1.**
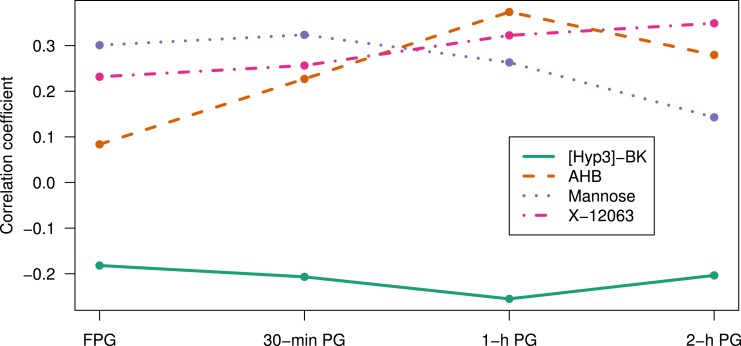
Correlation of metabolic markers with PG measurements. The metabolic markers correlated more significantly with 30-min PG and 1-h PG than with FPG. [Hyp3]-BK (green continuous line) and AHB (red dashed line) showed the strongest correlation with 1-h PG, mannose (blue dotted line) with 30-min PG, and X-12063 (pink dotted-dash line) with 2-h PG. The *α*-tocopherol and 10:1 carnitine showed low correlations with the PG measurements and are not shown in the figure.

Mannose showed higher correlation with the FPG (Pearson correlation coefficient, *r* = 0.30) and 30-minute PG (*r* = 0.32) than with 1-hour PG (*r* = 0.26) or 2-hour PG (*r* = 0.14). AHB correlated poorly with FPG (*r* = 0.08) but more strongly with post-OGTT PG values, showing the strongest correlation with 1-hour PG (*r* = 0.37). [Hyp3]-BK showed the strongest correlation with 1-hour PG (*r* = −0.25) and X-12063 with 2-hour PG (*r* = 0.35). In contrast, *α*-tocopherol and 10:1 carnitine were not associated with PG measurements (Fig. 1). HbA1c showed higher correlation with mannose (*r* = 0.16) and X-12063 (*r* = 0.16) than with other metabolites [Table D3 in (20)]. On average, metabolites showed higher correlation with PG values than with HbA1c.

### Predictive models

We used a regularized least squares regression approach to build predictive models for incident type 2 diabetes by using various combinations of PG (FPG, 30-minute PG, 1-hour PG, or 2-hour PG) and INS levels (fasting INS, 30-minute INS, 1-hour INS, and 2-hour INS), HbA1c, metabolic markers, and clinical risk factors (age, sex, BMI, and FH), such as (1) combinations of PG or INS levels (2), combinations of PG levels and common clinical risk factors (3), combinations of metabolites (4), combinations of PG levels and metabolites, and (5) combinations of PG levels, HbA1c, clinical risk factors, and metabolites. Altogether, we evaluated 1527 predictive models whose predictive performances are summarized below [see also Tables D4–D10 in (20)].

### Predictive models including glucose measurements

**Figure 2.**
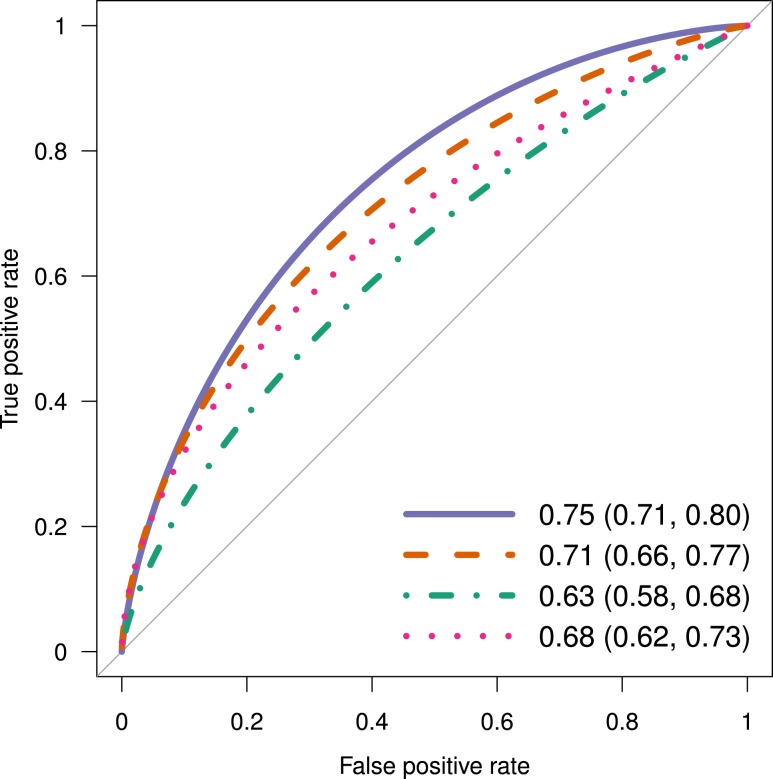
Comparison of predictive performances by PG measurements: FPG (green dotted-dash line; AUC 0.63; 95% CI, 0.58 to 0.69), 30-min PG (red dashed line; AUC 0.71; 95% CI, 0.66 to 0.77), 1-h PG (blue continuous line; AUC 0.75; 95% CI, 0.71 to 0.80), and 2-h PG (pink dotted line; AUC 0.68; 95% CI, 0.62 to 0.73). Among the PG measurements, 1-h PG predicted the incident type 2 diabetes the best followed by 30-min PG (*q* = 0.02 vs FPG, *q* = 0.12 vs 30-min PG, and *q* = 0.03 vs 2-h PG).

Among PG measurements, 1-hour PG showed the best predictive performance for type 2 diabetes, followed by 30-minute PG (Fig. 2). The 1-hour PG (AUC = 0.75, sensitivity = 0.75, specificity = 0.68) and 30-minute PG (AUC = 0.71, sensitivity = 0.62, specificity = 0.71) predicted progression to type 2 diabetes more accurately than FPG (AUC = 0.63, sensitivity = 0.55, specificity = 0.64) or 2-hour PG (AUC = 0.68, sensitivity = 0.56, specificity = 0.73). None of the PG pairs improved predictive performance beyond the 1-hour PG alone [Table D4 in (20)]. For instance, the combined model with 30-minute PG and 1-hour PG (AUC = 0.76, sensitivity = 0.72, specificity = 0.66) or FPG and 2-hour PG (AUC = 0.70, sensitivity = 0.59, specificity = 0.71) performed only as well as the model with 1-hour PG alone (false discovery rate *q*-value = 0.42 and 0.07, respectively). The combined model with all four PG values (AUC = 0.77, sensitivity = 0.73, specificity = 0.69, *q* = 0.31) did not improve prediction beyond 1-hour PG alone.

### Predictive models including clinical risk factors

The model with only the clinical risk factors, age, sex, BMI, and FH (AUC = 0.66, sensitivity = 0.69, specificity = 0.55) was clearly worse than 1-hour PG alone (*q* = 0.02). Both 30-minute PG and 1-hour PG, but neither FPG nor 2-hour PG, significantly improved the predictive performance of the clinical risk factor model. In particular, combining 1-hour PG and clinical risk factors (AUC = 0.77, sensitivity = 0.77, specificity = 0.70) improved the predictive performance of the clinical model (*q* = 0.02), but not that of 1-hour PG (*q* = 0.45).

### Predictive models including HbA1c

The model with HbA1c, although showing lower performance than 1-hour PG (AUC = 0.67, sensitivity = 0.65, specificity = 0.64, DeLong *p*-value = 0.03), was not significantly worse after accounting for multiple hypothesis testing (*q* = 0.09). Combining HbA1c with clinical risk factors or any combination of PG values did not outperform 1-hour PG alone. In particular, the combination of HbA1c with clinical risk factors (AUC = 0.71, sensitivity = 0.68, specificity = 0.67, *q* = 0.33), FPG (AUC = 0.70, sensitivity = 0.60, specificity = 0.71, *q* = 0.20), or both (AUC = 0.72, sensitivity = 0.55, specificity = 0.80, *q* = 0.34) resulted in models comparable with 1-hour PG. The combination of HbA1c and 1-hour PG (AUC = 0.76, sensitivity = 0.78, specificity = 0.68) was also comparable to 1-hour PG alone (*q* = 0.31) but significantly outperformed HbA1c alone (*q* = 0.02).

### Predictive models including metabolic markers

We evaluated the predictive performance of single metabolites and combinations of two, three, four, five, or all six metabolites [Table D5 in (20)]. Mannose was not significantly worse than 1-hour PG (AUC = 0.70, sensitivity = 0.60, specificity = 0.72, *q* = 0.16), but 1-hour PG was superior to every other metabolite. The 1-hour PG significantly outperformed six multivariate models including pairs of metabolites, and one model including three metabolites (*q* < 0.05). In contrast, no combination of metabolites significantly outperformed 1-hour PG alone. The multivariate model containing all six metabolites was comparable with 1-hour PG (AUC = 0.78, sensitivity = 0.67, specificity = 0.75, *q* = 0.27). Combining clinical risk factors with metabolites did not improve the predictive performance beyond 1-hour PG. The multivariate model including HbA1c and six metabolic markers (AUC = 0.82, sensitivity = 0.81, specificity = 0.72) outperformed 1-hour PG alone (*q* = 0.04).

### Predictive models including FPG and metabolic markers

In combination with FPG, no single metabolic marker outperformed 1-hour PG significantly, nor was the combination of FPG and the six metabolic markers (AUC = 0.78, sensitivity = 0.77, specificity = 0.69) significantly better than 1-hour PG (*q* = 0.21). Adding clinical risk factors to this model did not improve the model beyond 1-hour PG either (AUC = 0.78, sensitivity = 0.71, specificity = 0.76, *q* = 0.40). Furthermore, no combination of FPG, clinical risk factors, and metabolic markers was significantly better than 1-hour PG. However, the combination of HbA1c, FPG, and six metabolic markers outperformed 1-hour PG alone (AUC = 0.82, sensitivity = 0.79, specificity = 0.71, *q* = 0.03). The combination of HbA1c, FPG, and five metabolites—AHB, mannose, *α*-tocopherol, X-12063, and 10:1 carnitine—also outperformed 1-hour PG (AUC = 82, sensitivity = 0.76, specificity = 0.74, *q* = 0.04). No other combination involving HbA1c, FPG, clinical markers, and metabolic markers significantly outperformed 1-hour PG [Table D6 in (20)].

### Predictive models including 30-minute PG and metabolic markers

Twenty-four multivariate models involving the combination of HbA1c and 30-minute PG with metabolic markers outperformed 1-hour PG alone [Table D7 in (20)]. All of these included HbA1c and at least three metabolites, and four models included clinical risk factors. For instance, the combination of HbA1c, 30-minute PG, and six metabolites outperformed 1-hour PG (AUC = 0.84, sensitivity = 0.75, specificity = 0.79, *q* = 0.02).

### Predictive models including 1-hour PG and metabolic markers

All combinations of 1-hour PG, HbA1c, metabolic markers, and clinical risk factors evaluated (n = 252) showed higher AUC than 1-hour PG alone [*ie,* AUC > 0.75) [Table D8 in (20)]. Of these, 109 models significantly outperformed 1-hour PG (*ie,q<* 0.05). Among the 109 models, 42 models included 1-hour PG and metabolites, 49 models included 1-hour PG, HbA1c, and metabolites, two models included 1-hour PG, clinical risk factors, and metabolites, and 16 models included 1-hour PG, HbA1c, clinical risk factors, and metabolites.

**Figure 3.**
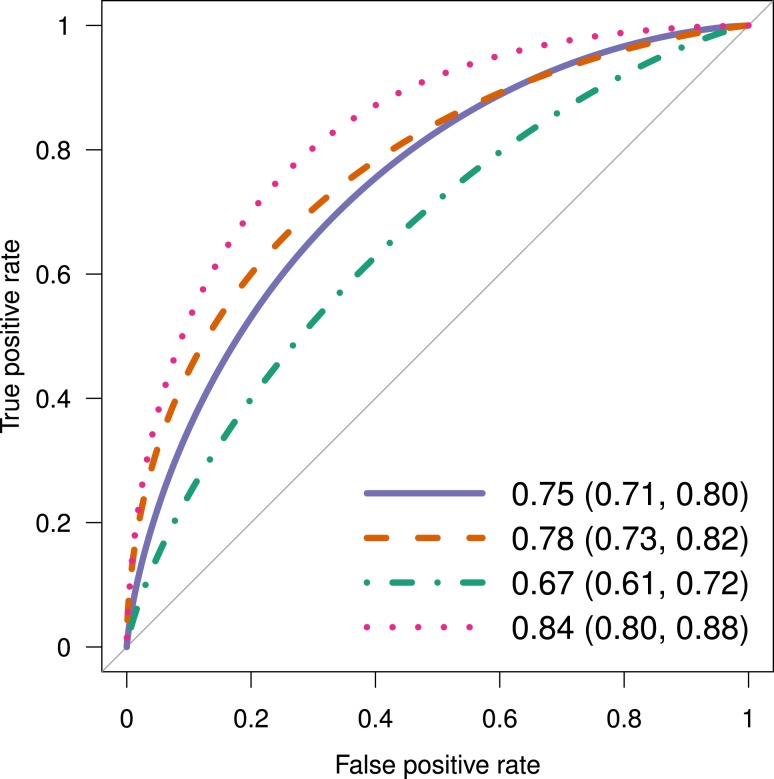
Comparison of predictive performances between 1-h PG, six metabolic markers, HbA1c, and their combination. Prediction of progression to type 2 diabetes with 1-h PG, HbA1c, and metabolic markers (mannose, AHB, [Hyp3]-BK, *α*-tocopherol, X-12063, and 10:1 carnitine). ROC curve of the predictive model with 1-h PG alone (blue continuous line; AUC 0.75; 95% CI, 0.71 to 0.80), the model with the six metabolic markers (red dashed line; AUC 0.78; 95% CI, 0.73 to 0.82), the model with HbA1c alone (green dotted-dash line; AUC 0.67; 95% CI, 0.61 to 0.72), and the model with 1-h PG, HbA1c, and the six metabolic markers (pink dotted line; AUC 0.84; 95% CI, 0.80 to 0.88) are shown. The model with the metabolic markers was slightly but not significantly better than the model with 1-h PG alone (*q* = 0.27). The combined model with 1-h PG, HbA1c, and the metabolic markers was significantly better than the model with 1-h PG alone (*q* = 0.02) or with HbA1c alone (*q* = 0.02).

The combined model with 1-hour PG and all six metabolic markers (AUC = 0.82, sensitivity = 0.79, specificity = 0.77) outperformed 1-hour PG alone (*q* = 0.02). The combined model with HbA1c, 1-hour PG, and six metabolic markers (AUC = 0.84, sensitivity = 0.73, specificity = 0.82) also outperformed 1-hour PG (*q* = 0.02; Fig. 3). Similarly, the combined model with clinical risk factors, HbA1c, 1-hour PG, and six metabolic markers (AUC = 0.83, sensitivity = 0.84, specificity = 0.73) also outperformed 1-hour PG (*q* = 0.03).

### Predictive models including 2-hour PG and metabolic markers

Three multivariate models including HbA1c, 2-hour PG, and metabolic markers significantly outperformed 1-hour PG alone. For instance, the combination of HbA1c, 2-hour PG, and six metabolic markers outperformed 1-hour PG (AUC = 0.82, sensitivity = 0.72, 0.80, *q* = 0.03). Two other models that included HbA1c, 2-hour PG, and five metabolites outperformed 1-hour PG [Table D9 in (20)].

### Predictive models including INS measurements

The predictive performance of 1-hour PG was superior to that of fasting INS (*q* = 0.02), 30-minute INS (*q* = 0.02), 1-hour INS (*q* = 0.02), 2-hour INS (*q* = 0.03), their pairwise combinations, or their combination with clinical risk factors [Table D10 in (20)]. On the other hand, 67 out of 220 models that included INS variables showed higher AUC than 1-hour PG but did not provide significant improvement.

### Improvement of predictive performance due to 1-hour PG or HbA1c

We evaluated whether adding 1-hour PG significantly improved the predictive performance of 252 multivariate models involving combinations of HbA1c, clinical risk factors, and metabolic markers. The addition of 1-hour PG improved the predictive performance of 184 models, in particular every combination of metabolites with or without the clinical risk factors [Table D11 in (20)]. For instance, the combination of 1-hour PG and six metabolites outperformed combination of six metabolites (*q* = 0.01). Of 252 models evaluated, however, 1-hour PG did not significantly improve the performance of 68 models [Table D11 in (20)], which involved either a combination of HbA1c and clinical risk factors (n = 55) or just HbA1c (n = 13) with metabolic markers. In contrast, addition of HbA1c alone did not significantly improve the performance of any of the models [Table D12 in (20)].

### Predictive models in individuals with IFG or IGT at baseline

Among the 543 participants in our study, 324 had neither IFG nor IGT at the beginning of the study (called IFG/IGT-free group), and 198 participants had either IFG or IGT (called IFG/IGT group) [Table D1 in (20)]. To verify whether our results are valid independent of IFG or IGT status at baseline, we tested whether the predictive performance of postload PG values follows the same trend as in the entire population and whether combining metabolic markers would improve the predictive performance of 1-hour PG or HbA1c.

The 1-hour PG outperformed FPG (*P* = 0014) and 2-hour PG (*P* = 0.016) and was slightly but not significantly better than 30-minute PG (*P* = 0.46) or HbA1c (*P* = 0.09) in predicting the future progression to type 2 diabetes in the IFG/IGT-free group [Fig. D1 in (20)]. However, in the IFG/IGT group 1-hour PG outperformed FPG (*P* = 0.006) and was comparable to 30-minute PG (*P* = 0.05), 2-hour PG (*P* = 0.12), and HbA1c (*P* = 0.62) [Fig. D3 in (20)].

The multivariate model with metabolic markers alone was comparable to 1-hour PG alone in the IFG/IGT-free group (*P* = 0.07) and in the IFG/IGT group (*P* = 0.72). The multivariate model combining the 1-hour PG, HbA1c, and metabolic markers was significantly better than 1-hour PG (*P* = 0.01) and HbA1c (*P* = 0.001) in the IFG/IGT-free [Fig. D2 in (20)] group as well as in the IFG/IGT group (*P* = 0.004 and 0.0006, respectively) [Fig. D4 in (20)].

## Discussion

The FPG and 2-hour PG after a 75-g OGTT have traditionally been used to identify people at risk for developing type 2 diabetes. More recently, 1-hour PG level ≥8.6 mmol/L has been shown to be a more sensitive biomarker of dysglycemia than FPG, 2-hour PG, or HbA1c (2). On the other hand, metabolomics has provided a rich source of biomarkers for the prediction of type 2 diabetes (16–18). The aim of this study was to provide a systematic assessment of the joint predictive performance of metabolic markers and PG levels measured at different time points during the OGTT. We constructed machine learning predictive models based on combinations of PG and INS values, other clinical risk factors, HbA1c, and metabolic markers that we previously reported (18), thereby evaluating the performance of 1527 models.

Consistent with previous reports, 1-hour PG alone showed superior predictive performance (AUC = 0.75, sensitivity = 0.75, specificity = 0.68) to clinical risk factors (false discovery rate *q-*value = 0.02), FPG (*q* = 0.02), and 2-hour PG (*q* = 0.03) but not 30-minute PG (*q* = 0.12) or HbA1c (*q* = 0.09). Based on an analysis of 1949 participants from the BPS, of whom 132 progressed to type 2 diabetes during an average follow-up period of 4.94 years, Alyass *et al.* (3) showed that 1-hour PG outperformed HbA1c in predicting the future progression to type 2 diabetes. Our results indicate that the difference between 1-hour PG and HbA1c was marginal (*q* = 0.09). However, with regard to the comparison between 1-hour PG and HbA1c, the difference between our analysis and that of Alyass *et al.* (3) is due to the simultaneous comparison of 1526 predictive models with 1-hour PG in our study, which required an assessment of the statistical difference in light of multiple testing. The *P* value derived from the DeLong test of ROC curves between 1-hour PG and HbA1c in our study is 0.03, which would have been deemed a significant difference in the absence of an exhaustive evaluation of a large number of competing predictive models. Alyass *et al.* (3) observed an AUC of 0.80 (95% CI, 0.77 to 0.84) for 1-hour PG in the BPS cohort and an AUC of 0.70 (95% CI, 0.68 to 0.73) in the Malmö Preventive Project (MPP) study, in which the follow-up period was 23.5 years. They therefore postulated that 1-hour PG may better predict the type 2 diabetes risk in the short term. Our result for 1-hour PG with an AUC of 0.75 (95% CI, 0.71 to 0.80) during the 10-year follow-up period appears to be in agreement with the hypothesis. The AUC derived from HbA1c in our study, 0.67 (95% CI, 0.61 to 0.72), is comparable to the AUC of 0.69 (95% CI, 0.64 to 0.74) Alyass *et al.* (3) derived for HbA1c. Thus, we believe that our results are in agreement with those of Alyass *et al.* (3), and the statistical discrepancy concerning the difference between HbA1c and 1-hour PG is primarily a result of the difference in the number of hypotheses tested and secondarily may stem from the differences in the number of samples and the follow-up period. Abdul-Ghani *et al.* (27) previously showed that although HbA1c is a weaker predictor of progression to type 2 diabetes than 1-hour PG, combining HbA1c and 1-hour PG together improves the predictive performance of the latter. Our results showed that the combination of HbA1c and 1-hour PG was significantly better than HbA1c (*q* = 0.02) but not 1-hour PG (*q* = 0.31).

However, the results of the current study indicate that HbA1c is indeed a robust predictor of progression to type 2 diabetes in combination with clinical risk factors, PG measurements, and metabolic markers. For instance, although the predictive models including clinical risk factors alone and FPG alone both clearly showed lower performance than 1-hour PG (*ie*, *q* < 0.05), in combination with HbA1c both the models became comparable to 1-hour PG (*ie*, *q* ≥ 0.05). Second, the difference between 1-hour PG and HbA1c was insignificant in the IFG/IGT and IFG/IGT-free groups, although the one-to-one DeLong *P*-value in the whole population was 0.03. Third, among the 139 predictive models that outperformed 1-hour PG, 95 models contained HbA1c as a variable. Moreover, the combination of HbA1c alone with six metabolic markers (without any other PG variable) outperformed 1-hour PG.

Of all 1527 predictive models evaluated in this study, 139 models outperformed 1-hour PG in terms of AUC (*q* < 0.05). All these models included at least one metabolite as a variable. Besides the metabolites, the addition of HbA1c alone, 1-hour PG alone, 1-hour PG and clinical risk factors, or HbA1c and a PG measurement was necessary to achieve a higher predictive performance than 1-hour PG alone. The 1-hour PG significantly improved predictive performance of all multivariate models of metabolites [Table D11 in (20)], whereas HbA1c did not [Table D12 in (20)].

Although the DeLong test allows a formal comparison of the ROC curves, other statistics such as sensitivity and specificity are more useful in clinical practice. The 1-hour PG showed a good balance between sensitivity (0.75) and specificity (0.68). Of the 139 predictive models that outperformed 1-hour PG in terms of DeLong comparison of the ROC curves, 74 showed greater sensitivity than 1-hour PG, 132 showed greater specificity, 68 showed both greater sensitivity and specificity, and 47 showed greater sensitivity, specificity, accuracy, PPV, and NPV than 1-hour PG. Among these 47 models [Table D13 in (20)] were the multivariate model including HbA1c and the panel of six metabolites (AHB, [Hyp3]-BK, mannose, *α*-tocopherol, 10:1 carnitine, and X-12063); the model including HbA1c, FPG, and six metabolites; and 10 different combinations of HbA1c and 30-minute PG with metabolites; 34 multivariate models that included the combination of 1-hour PG and metabolites with or without HbA1c and clinical risk factors; and a multivariate model including HbA1c, 2-hour PG, and five metabolites. HbA1c was included in 28 of these models, and clinical risk factors were included in six models.

The model including 1-hour PG and the panel of six metabolites was among the 47 models that improved all predictive performance measures over 1-hour PG. The sparsest of the 47 models included 1-hour PG and mannose. Moreover, mannose was included in the highest number of models (44 out of 47). Thus, mannose may be a crucial metabolic marker in combination with other predictors such as 1-hour PG for assessing risk of type 2 diabetes. Mannose, produced after the phosphorylation of glucose, is thought to be tightly linked to the metabolism of glucose and fructose. Mannose alone was comparable to 1-hour PG (*q* = 0.16). The potential of mannose as a biomarker for type 2 diabetes has also been shown previously (28, 29). However, routine use of mannose or other metabolites for assessing the risk of type 2 diabetes may not be possible in a clinical setting until cost-effective assays become available.

Mannose correlated highly with FPG (*r* = 0.30) and 30-minute PG (*r* = 0.32). The other metabolites showed higher correlation with post-OGTT PG values than with FPG. The most striking example was AHB, which correlated poorly with FPG (*r* = 0.08) but more strongly with 1-hour PG (*r* = 0.37). [Hyp3]-BK showed the strongest negative correlation with 1-hour PG (*r* = −0.25), whereas X-12063 correlated the strongest with 2-hour PG (*r* = 0.35). The fact that the majority of metabolic markers were more strongly correlated with postload glucose values than with FPG suggests that metabolic markers reflect stimulated (glucose) metabolism, unlike FPG (30). On average, the metabolic marker panel showed the highest correlation with 1-hour PG. The four metabolic markers that highly correlated with 1-hour PG (*ie*, mannose, AHB, [Hyp3]-BK, and X-12063) also predicted progression to type 2 diabetes with similar predictive performance as 1-hour PG (AUC = 0.75, sensitivity = 0.68, specificity = 0.71, *q* = 0.53). These results indicate that the metabolic marker signature may capture key changes associated with response to glucose stimulation. In accordance with these findings, AHB and X-12063 have previously been shown to be biomarkers of impaired glucose tolerance (31, 32). Ho *et al.* (33) showed that AHB was among the metabolites altered in response to OGTT. Other studies have reported AHB, mannose, and X-12063 as predictive markers of type 2 diabetes (22, 28, 29, 32), and our results showed that the combination of 1-hour PG, AHB, mannose, and X-12063 (AUC = 0.80, sensitivity = 0.76, specificity = 0.75) improved the predictive performance compared with the combination of AHB, mannose, and X-12063 (*q* = 0.01) and 1-hour PG (*q* = 0.03).

We provide strong evidence that 1-hour PG is a robust predictor of future type 2 diabetes, together with other reports based on large-scale epidemiologic trials in different populations. The 1-hour PG significantly outperformed every individual marker except for 30-minute PG, HbA1c, or mannose, which were statistically comparable, albeit with lower AUC and sensitivity. Adding metabolites improves the predictive value of 1-hour PG but significantly adds to measurement costs. Nevertheless, our results show that metabolic markers are robust predictors in combination with PG measurements or HbA1c. In particular, mannose was identified as a crucial marker. It is possible that metabolites could further improve the predictive value of diabetic subgroups, because we have recently shown that type 2 diabetes can be subdivided into five subgroups with six clustering variables (age at diagnosis, BMI, HbA1c, GAD autoantibodies, homeostatic model assessment of β-cell function, and homeostatic model assessment of INS resistance) with C-peptide measurements (34). The predictive accuracy may also be further improved by including additional anthropometric and lifestyle data (35).

### Limitations

The size of our study population (n = 543) diminishes when divided into IFG/IGT (n = 198) and IFG/IGT-free groups (n = 324), thus making it difficult to robustly evaluate whether our findings hold true independent of the IFG and IGT status at baseline. However, the predictive performances of the models we evaluated showed similar trends as in the whole study population. Replication of our results in an independent study would increase the value of our findings, but we were unable to identify any study with all the measurements needed (*ie*, clinical risk factors, HbA1c, PG, and INS measurements, as well as the metabolic markers). However, our findings related to the predictive performance of 1-hour PG have been identified by other large-scale studies, as discussed in this article, and we hope that our study forms a basis for future validation studies by other researchers.

## Conclusions

In summary, 1-hour PG is an effective biomarker for predicting future type 2 diabetes either alone or in combination with metabolic markers. The 30-minute PG, HbA1c, and mannose are statistically comparable to 1-hour PG, although they show slightly lower performance. Metabolic markers provide a robust prediction of future risk of type 2 diabetes in combination with postload PG measurements and HbA1c. Shortening the standard 75-g OGTT to 1 hour improves its predictive value and clinical usability. International diabetes organizations should consider this recommendation because 1-hour PG more accurately identifies patients with dysglycemia, critical in context of the burgeoning global population with obesity and glucose disorders.

## Abbreviations:

AHB: *α*-hydroxybutyrate
AUC: area under the receiver operating characteristic curve
BMI: body mass index
BPS: Botnia Prospective Study
FH: familial history
FPG: fasting plasma glucose
HbA1c: glycated hemoglobin
[Hyp3]-BK: bradykinin-hydroxyproline
IFG: impaired fasting glucose
IGT: impaired glucose tolerance
INS: insulin
NPV: negative predictive value
OGTT: oral glucose tolerance test
PG: plasma glucose
PPV: positive predictive value
ROC: receiver operating characteristic
